# Src/Fas2-dependent Ephrin phosphorylation initiates Eph/Ephrin reverse signaling through Rac1 to shape columnar units in the fly brain

**DOI:** 10.1126/sciadv.adv7490

**Published:** 2025-08-13

**Authors:** Miaoxing Wang, Xujun Han, Yunfei Lee, Rie Takayama, Makoto Sato

**Affiliations:** ^1^ Mathematical Neuroscience Unit, Institute for Frontier Science Initiative, Kanazawa University, Kanazawa, Ishikawa, Japan.; ^2^ Graduate School of Medical Sciences, Kanazawa University, Kanazawa, Japan.; ^3^ Laboratory of Developmental Neurobiology, Graduate School of Medical Sciences, Kanazawa University, Kanazawa, Ishikawa, Japan.

## Abstract

Columns are the morphological and functional units containing multiple neurons in the brain. The molecular mechanisms of column formation are largely unknown. Ephrin/Eph signaling mediates a variety of developmental processes. Ephrin acts as a ligand for Eph to regulate forward signaling, whereas Eph acts as a ligand for Ephrin to regulate reverse signaling. However, whether and how the uni- or bidirectional Ephrin/Eph signaling is involved in column formation remains elusive. In this study, we show that Ephrin and Eph regulate the morphology and location of columnar neurons through bidirectional repulsive signaling. Furthermore, Eph ligand triggers cytoplasmic tyrosine phosphorylation of Ephrin under the control of Src kinases and Fasciclin 2 (Fas2), forming the Ephrin/Src/Fas2 complex to promote reverse signaling through a downstream regulator, Rac1. This study provides the detailed analysis of the molecular interactions involved in column formation using the fly brain as a model.

## Teaser

Reverse Ephrin signaling drives fly brain column formation—mechanistic link via Src/Fas2-Rac1.

## INTRODUCTION

In various brain tissues, columns are the structural and functional units containing multiple neurons. Within a column, the morphology and projection of diverse neurons are highly organized to form precise neural circuits. However, the developmental mechanism of columnar unit formation remains largely unknown.

The brain of *Drosophila melanogaster* is an excellent model for exploring the genetic and molecular mechanisms of column formation as it shares similar columnar structures with the mammalian brain with fewer neurons included (*1*–*3*). The fly visual system includes the retina and the optic lobe, which consists of the lamina, medulla, and lobula complex. The medulla, the largest neuropil of the optic lobe, contains ~100 morphologically distinct types of neurons forming 800 columns (*4*). Visual information received by photoreceptors in the retina is relayed through the lamina to the distal medulla. R7 and R8 photoreceptors and L1 to L5 lamina neurons directly innervate the medulla neuropil, and it is known that the growth cones of R7, R8, and L1 to L5 neurons compose a single medulla column together with the other columnar medulla neurons (*5*). Cell adhesion molecules such as N-cadherin (Ncad) and Flamingo (Fmi) play important roles in the process of guidance and rearrangement of the growth cones of R7, R8, and lamina neurons (*6*–*12*).

**Fig. 1.**
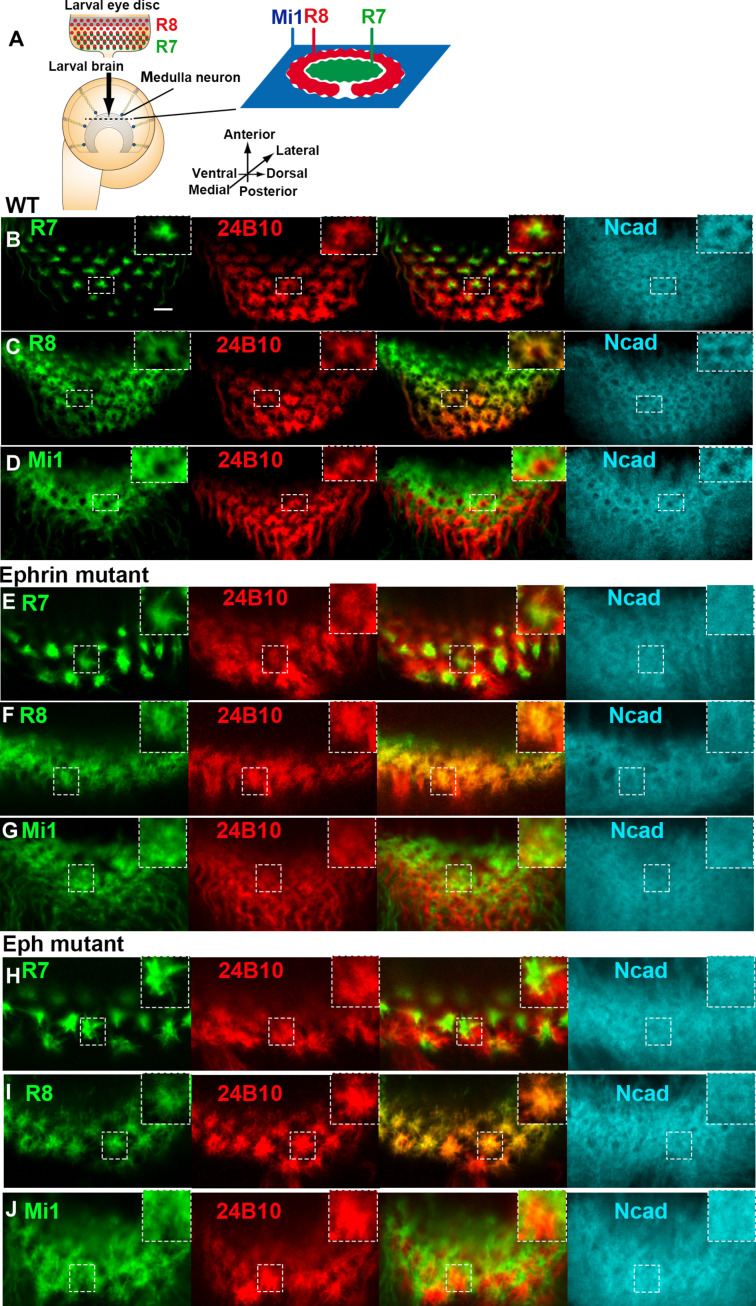
Ephrin and Eph regulate the morphology of neurites of columnar neurons. Schematics of the developing larval visual systems. In the larval eye disc, R8 and R7 are sequentially differentiated behind the morphogenetic furrow. Columns are identifiable along the planes indicated by dashed lines in the optic lobe. (**A** to **D**) R7 growth cone (B; green) occupies the dot-like central region of the column shown by Ncad (blue), R8 (C; green) enwraps the R7 growth cone forming a horseshoe-like region, and Mi1 (D; green) occupies a grid-like region outside the R8 growth cone. 24B10 (red) visualizes R8 growth cones. (**E** to **J**) In *Ephrin*-null mutant (*Ephrin^I95^*) (E to G) and *Eph*-null mutant (*Eph^X652^*) (H to J), the morphology of the three core columnar neurons is disorganized: R7 shows an expanded axon growth cone; R8 axon growth cone penetrates the central hole region, disrupting the horseshoe-like pattern; Mi1 neurite also penetrates the central hole region, disrupting the grid-like pattern. Scale bar, 5 μm.

The medulla intrinsic neuron Mi1 is a unicolumnar neuron, projecting to the peripheral edge region of the columns during larval development. Our previous study demonstrated that R7, R8, and Mi1 are the core columnar neurons that are concentrically arranged in the larval medulla according to Ncad-dependent differential adhesion (*3*). The growth cone of R7 occupies the dot-like central region of the column. The R8 growth cone enwraps the R7 growth cone forming a horseshoe-like region, and the Mi1 growth cone occupies a grid-like region outside the R8 growth cone (Fig. 1, A to D). The precise organization of these columnar neurons is regulated by multiple molecules (*3*, *13*–*15*).

Ephrin and Eph have been shown to be involved in not only diverse developmental processes but also a variety of diseases (*16*–*20*). They can both act as ligands and receptors for forward and reverse signaling through cell-to-cell communication (*21*–*24*). In vertebrates, they are divided into two main subfamilies of ligand/receptor couples, Ephrin-A/Eph-A and Ephrin-B/Eph-B, based on their specific structures (*25*, *26*). Ephrin-B reverse signaling has been shown to have multiple functions in various developmental processes that are dependent on or independent from its cytoplasmic tyrosine phosphorylation. Three of five tyrosines conserved in the Ephrin-B subfamily have been characterized as the major phosphorylation sites in vitro (*27*). It has been reported that Src family kinases (SFKs) are positive regulators of Ephrin-B phosphorylation (*28*). However, these studies are essentially based on cultured cells and/or in vitro systems. The molecular mechanism and biological significance of Ephrin phosphorylation in vivo remain elusive.

Ephrin-A and Ephrin-B have been shown to be involved in column formation by integrating the distribution of cortical pyramidal neurons at a macroscopic level (*29*, *30*). In the fly visual system, the Ephrin homolog related to Ephrin-B is required for topographic map formation and branching restriction of lamina neurons (*31*–*33*). However, the involvement of uni- or bidirectional Ephrin/Eph signaling in column formation, and how it functions, remains elusive.

In this study, we demonstrate that Ephrin and Eph regulate the morphology and location of core columnar neurons during column formation through bidirectional repulsive signaling in vivo using the fly brain as a model. We generated Ephrin antibodies that specifically detect phosphorylated and unphosphorylated forms of its intracellular domain. Using these antibodies, we found that they show different distribution patterns complementary with each other in the core columnar neurons. Phosphorylated Ephrin is specifically localized in R7 showing a dot-like pattern, whereas unphosphorylated Ephrin and Eph are localized more widely in R8 and Mil showing a grid-like expression pattern. They regulate the morphology and segregation of the core neuron growth cones by the bidirectional repulsive activity of Ephrin/Eph signaling through interaction between the columnar neurons. Ephrin expressed in R7 is phosphorylated on tyrosine residues in the cytoplasmic domain and is regulated by Src kinases and the *Drosophila* ortholog of neural cell adhesion molecule (NCAM), Fasciclin 2 (Fas2), under the control of Eph expressed in R8. Furthermore, we also found that Rac1 acts as a downstream effector of Eph/Ephrin reverse signaling. Thus, this study presented a comprehensive analysis of the complex molecular interactions in the defined context of column formation.

## RESULTS

### Ephrin/Eph signaling is required for columnar neuron organization

To investigate how *Ephrin* and *Eph* function in column formation, we first observed the morphology and organization of the core columnar neurons in *Ephrin* and *Eph*-null mutants. Because column formation is initiated by three core neurons, R7, R8, and Mi1, establishing distinct concentric domains within a column (Fig. 1A), we focus on the larval medulla as a model system to examine the roles of *Ephrin* and *Eph* in column formation. Under wild-type conditions, the R7 growth cone localizes to the dot-like central region of the column, the R8 growth cone envelops the R7, forming a horseshoe-like region, and the Mi1 growth cone extends to occupy the grid-like area surrounding the R8 (Fig. 1, A to D). In the *Ephrin*-null mutant (*Ephrin^I95^*), the morphology of all these three core columnar neurons was disorganized: The R7 axon exhibited an expanded growth cone; the R8 axon growth cone invaded the central region, disrupting the horseshoe-like structure; and the Mi1 neurite extended into the central region, compromising the grid-like arrangement (Fig. 1, E to G). A similar phenotype was observed in the *Eph*-null mutant (*Eph^X652^*; Fig. 1, H to J), suggesting that both *Ephrin* and *Eph* are crucial for organizing these columnar neurons. The observed reduction in the number of R7, R8, and Mi1 growth cones in the third larva and later stages suggests that Ephrin and Eph signaling likely play a role in regulating their projections to the medulla neuropil.

We further examined the phenotype in the adult brain. Because there are numerous sequential events influenced by multiple neurons during the pupal stages as described in the previous literature (*6*, *7*), the final phenotype is substantially disorganized and difficult to directly correlate with the larval phenotype. Therefore, in this study, we focus only on the interaction between their growth cones during the larval stage.

### Phosphorylated Ephrin and unphosphorylated Ephrin show distinct distribution patterns

**Fig. 2.**
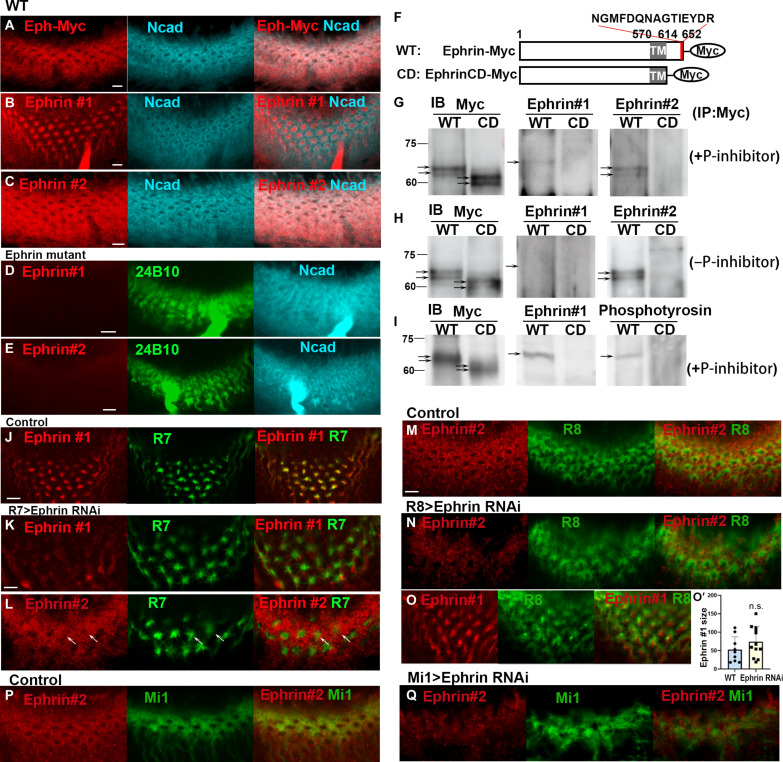
Distributions of Eph and Ephrin in columnar neurons. (**A**) Anti-Myc antibody staining revealed a grid-like expression of Eph-Myc (red) overlapping with Ncad staining (blue). (**B** and **C**) Ephrin #1 and Ephrin #2 antibodies (red) show different patterns that are complementary to each other. Ephrin #1 shows a dot-like pattern, and Ephrin #2 shows a grid-like pattern. Ncad, blue. (**D** and **E**) Both the signals of Ephrin #1 and Ephrin #2 were lost in the *Ephrin* mutant. (**F**) Structure of Ephrin-Myc (WT) and EphrinCD-Myc (CD), a cytoplasmic deletion mutant. (**G** and **H**) Validation of Ephrin antibodies by immunoblotting (IB) followed by anti-Myc immunoprecipitation (IP). In the presence of phosphatase inhibitor, Ephrin #1 detects one band in WT, whereas Ephrin #2 detects two bands that are also detected with Myc-antibody in WT (G). In the absence of the phosphatase inhibitor, no band is detected by Ephrin #1, whereas signals of Ephrin #2 are enhanced (H). (**I**) Validation of Ephrin phosphorylation by immunoblotting followed by anti-Myc immunoprecipitation. In the presence of phosphatase inhibitor, both Ephrin #1 and phosphotyrosine antibodies detect one band in WT, which is absent in CD. (**J** to **L**) Knocking down *Ephrin* in R7 decreased Ephrin #1 signals and caused ectopic Ephrin #2 signals (arrows in L). (**M** to **O**) Ephrin knockdown in R8 (N) reduced Ephrin #2 signals (red) in R8 growth cones (*R8-Gal4 UAS-GFP*, green) without affecting Ephrin #1 signals (O). (**O′**) Quantification of the area size of Ephrin #1 signals (in J and O). (**P** and **Q**) Ephrin knockdown in Mi1 (Q) reduced Ephrin #2 signals (red; *Mi1-Gal4 UAS-GFP*, green). Scale bars, 5 μm.

Next, we investigated the expression patterns of Ephrin and Eph in the developing medulla. The expression of Eph was visualized using the *Myc* knock-in allele of *Eph* (*34*). Anti-Myc antibody staining revealed a grid-like expression of Eph-Myc at the late third instar larval stage (Fig. 2A) and a donut-like pattern at 24 and 48 hours after puparium formation (APF) overlapping with Ncad staining (fig. S1, A and B). To visualize Ephrin distribution, we generated antibodies that recognize the intracellular domain of *Drosophila* Ephrin (Fig. 2F). The two Ephrin antibodies named Ephrin #1 and Ephrin #2 show different patterns that are complementary with each other. Ephrin #1 shows a dot-like pattern similar to the pattern of R7 growth cones throughout development (Fig. 2B and fig. S1, C and D). In contrast, Ephrin #2 shows a grid-like pattern similar to the pattern of Eph-Myc expression (Fig. 2, A and C, and fig. S1, E and F). In *Ephrin* mutant brains, the signals of Ephrin #1 and Ephrin #2 were lost, suggesting that both antibodies recognize Ephrin protein (Fig. 2, D and E).

We further tested the specificity of these two antibodies through immunoprecipitation of Myc-tagged Ephrin proteins overexpressed in photoreceptor neurons under the control of *GMR-Gal4*. Although the Ephrin antibodies recognized the full-length Myc-tagged Ephrin (Ephrin-Myc), they did not recognize the truncated protein that lacks the cytoplasmic domain (EphrinCD-Myc; Fig. 2, F to H), suggesting that they both recognize the cytoplasmic domain of Ephrin. Ephrin-Myc detected by Ephrin #1 was larger than that detected by Ephrin #2 in the presence of phosphatase inhibitor (Fig. 2G). However, the former was lost whereas the latter was increased in the absence of phosphatase inhibitor, suggesting that Ephrin #1 recognizes the phosphorylated Ephrin whereas Ephrin #2 recognizes the unphosphorylated Ephrin (Fig. 2H). The faint band detected by Ephrin #1 is consistent with the fact that the Ephrin #1 signal is found only in a limited area in the brain compared with that of Ephrin #2 (Fig. 2, B, C, and G).

Furthermore, we attempted to directly assess Ephrin phosphorylation using an anti-phosphotyrosine antibody. We observed that the phosphotyrosine signal was markedly reduced in the lane corresponding to EphrinCD-Myc compared to full-length Ephrin-Myc (Fig. 2I). These results suggest that the cytoplasmic domain of Ephrin is tyrosine phosphorylated and that this phosphorylation is significantly reduced when the C-terminal region is removed.

Here, we assume that both phosphorylated Ephrin and unphosphorylated Ephrin exist in the presence of phosphatase inhibitor resulting in the detection of Ephrin-Myc by Ephrin #1 and #2 (Fig. 2G). In the absence of phosphatase inhibitor, unphosphorylated Ephrin-Myc recognized by Ephrin #2 may become dominant due to the removal of phosphates from Ephrin (Fig. 2H). All the results suggest that antibody #1 preferentially recognizes phosphorylated Ephrin, whereas antibody #2 binds to the unphosphorylated form. Although absolute specificity cannot be claimed, our observations indicate that the antibodies exhibit a high degree of preference, allowing us to differentiate between phosphorylated and unphosphorylated states effectively.

### Phosphorylated Ephrin#1 is located within R7 growth cones

**Fig. 3.**
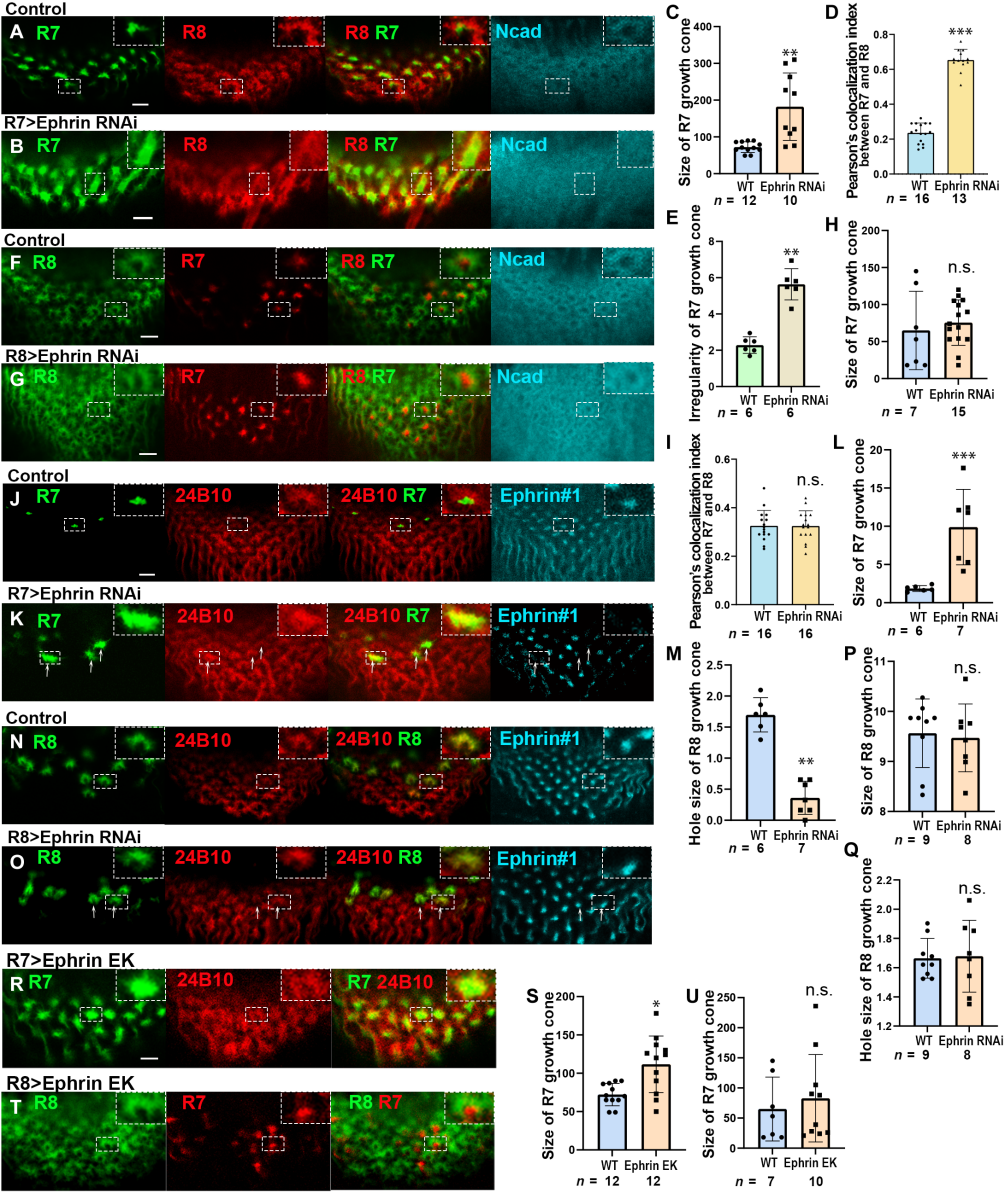
Ephrin expressed in R7 regulates the organization of columnar neurons. (**A** and **B**) *Ephrin* knockdown in R7 disturbs the separation of R7 (green) and R8 (red) growth cones and column formation (Ncad, blue). (**C**) Quantification of the size of R7 growth cones (in A and B). (**D**) Overlapping of the growth cones of R7 and R8 (in A and B). (**E**) Quantification of the R7 growth cone arrangement (in A and B). (**F** and **G**) *Ephrin* knockdown in R8 did not affect the separation of R7 (red) and R8 growth cones (green) and column formation (Ncad, blue). (**H**) Quantification of the size of R7 growth cones (in F, G). (**I**) Overlapping of the growth cones of R7 and R8 (in F and G). (**J** and **K**) R7-specific MARCM clones expressing *Ephrin* RNAi show the disorganization and overlapping of R7 (green) and R8 (24B10, red) growth cones. (**L**) Quantification of the size of R7 growth cones (in J and K). (**M**) Quantification of the hole size of R8 growth cones (in J and K). (**N** and **O**) R8-specific MARCM clones expressing *Ephrin* RNAi show no defects of R7 and R8 growth cones. Arrows indicate the clones. (**P**) Quantification of the size of R8 growth cones (in N and O). (**Q**) Quantification of the hole size of R8 growth cones (in N and O). (**R**) Overexpression of *EphrinEK* in R7 disturbs the separation of R7 (green) and R8 (24B10, red) growth cones. (**T**) Overexpression of *EphrinEK* in R8 did not affect the separation of R7 and R8 growth cones. (**S** and **U**) Quantification of the size of R7 growth cones (in R and T, respectively). The dashed boxes are enlarged in the top-right panels (in A, B, F, G, J, K, N, O, R, and T). Scale bars, 5 μm.

Ephrin #1 staining shows a dot-like pattern overlapping with R7 cell bodies in the eye and R7 axons in the lamina (fig. S2) as well as with R7 growth cones in the medulla (Fig. 2J). To investigate the source of phosphorylated Ephrin, we knocked down *Ephrin* specifically in R7 and found that the Ephrin #1 signal was down-regulated whereas R7 growth cones were enlarged (Fig. 2, J to K), suggesting that R7 is the source of phosphorylated Ephrin in the medulla. Note that ectopic Ephrin#2 signals were found in R7 growth cones under this condition (Fig. 2L), most likely due to the expansion of R8 as shown later (see Fig. 3, B and K).

Meanwhile, Ephrin #2 shows a grid-like pattern overlapping with R8 and Mi1. The Ephrin #2 signal was down-regulated when *Ephrin* was knocked down in R8 and Mi1 (Fig. 2, M, N, P, and Q), suggesting that R8 and Mi1 are the source of unphosphorylated Ephrin. However, the area showing Ephrin#1 signals at R7 growth cones were not significantly expanded under this condition (Fig. 2, O and O′). Together, Ephrin #1 detects the phosphorylated Ephrin in R7, and Ephrin#2 detects the unphosphorylated Ephrin in R8 and Mi1 in the medulla.

### Ephrin in R7 is autonomously and nonautonomously required for the organization of the columnar neurons

To investigate the function of *Ephrin* in organization of the column, we specifically knocked down *Ephrin* in R7, R8, and Mil neurons. When *Ephrin* was knocked down in R7, the morphology of R7 and R8 was affected (Fig. 3, A and B, and fig. S3, A and B). The growth cones of R7 were enlarged (Fig. 3, B and C, and fig. S3, B and C), and their arrangement became irregular (Fig. 3E). The morphology of R8 growth cones was disrupted, penetrating the central hole region and the neighboring columns (Fig. 3B). The overlap between R8 and R7 growth cones increased (Fig. 3D), and the column structure was severely disorganized as visualized by Ncad (Fig. 3, A and B). In addition, we generated R7-specific Mosaic Analysis with Repressive Cell Marker (MARCM) clones expressing Ephrin RNA interference (RNAi) (Fig. 3, J to M). The resulting phenotypes, including the expansion of R7 growth cones, disorganization of R8 growth cones, and significant overlap between these growth cones, were consistent with the effects observed in Ephrin RNAi knockdown in R7 cells (Fig. 3, A to I). Note that the specificity of Ephrin RNAi was verified in the previous rescue experiment (*34*). Because the R7 growth cone occasionally failed to reach the medulla, most likely due to the failure of the R7 projection, we focused only on the R7 growth cones found in the medulla.

To assess the final outcome of Ephrin knockdown, we examined adult columnar structures using *R7-Gal4 UAS-Ephrin RNAi* in combination with *Mi1-LexA LexAop-RFP* and conducted super-resolution imaging (Zeiss Airyscan). Note that *R7-Gal4* exhibits strong expression during the larval stage, but its activity becomes markedly reduced in early pupal stage and undetectable by the adult stage. Nevertheless, we observed clear morphological differences in Mi1 terminal arborizations between control and RNAi conditions (fig. S3, D to F). In the Ephrin RNAi condition, Mi1 terminals at the M10 layer displayed numerous elongated arborizations and frequent terminal fusions, indicative of disrupted columnar organization in the adult brain.

It has been reported that E320K mutation in the extracellular domain of Ephrin inhibits its binding to Eph in trans (*34*). Overexpression of *Ephrin^E320K^* (*Ephrin^EK^*) in R7 caused the defect in R7 and R8 growth cones, suggesting the directionality from R7 to R8 (Fig. 3, R and S). In contrast, overexpression of *Ephrin^EK^* in R8 caused no defect in either R8 or R7 (Fig. 3, T and U). Together, these results suggest that phosphorylated Ephrin in R7 regulates the morphology and separation of R7 and R8 growth cones.

In contrast, when *Ephrin* was knocked down in R8, the morphology of R7 and R8 was not significantly affected (Fig. 3, F to I), and the results of R8-specific Ephrin RNAi clones corroborated these observations (Fig. 3, N to Q). Because the columnar structure was disrupted compared to the control as visualized by Ncad (Fig. 3, F and G), other columnar neurons besides R7 and R8 might be affected. The morphology of Mi1 was disorganized under the same condition (fig. S4, A and B), indicating that unphosphorylated Ephrin in R8 regulates the morphology of Mi1 growth cones. When *Ephrin* was knocked down in Mi1, the morphology of R7, R8, and Mi1 was not significantly affected (fig. S4, C to L).

Because phosphorylated and unphosphorylated Ephrin are abundant in R7 and R8, respectively, and Eph is strongly localized to R8 and Mi1 neurites, the above results are consistent with the idea that phosphorylated Ephrin in R7 regulates column organization through Eph in R8 and unphosphorylated Ephrin in R8 regulates column organization through Eph in Mi1.

### Eph in R8 is autonomously and nonautonomously required for the organization of the columnar neurons

**Fig. 4.**
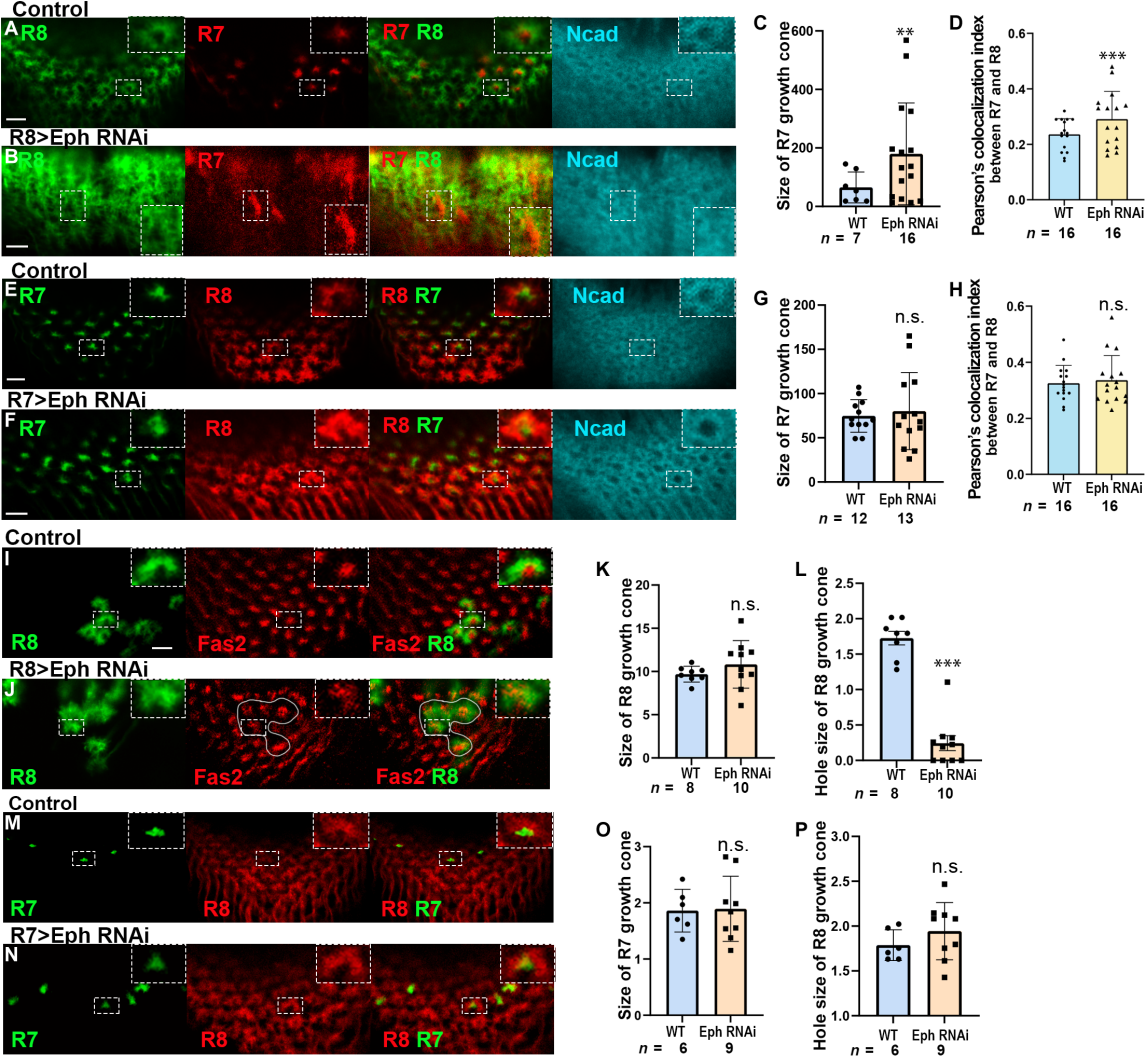
Eph expressed in R8 regulates the organization of columnar neurons. (**A** and **B**) *Eph* knockdown in R8 disturbs the separation of R8 (*R8-Gal4 UAS-GFP*, green) and R7 (*R7-LexA LexAop-RFP*, red) growth cones and column formation (Ncad, blue). (**C**) Quantification of the size of R7 growth cones (in A and B). (**D**) Overlapping of the growth cones of R7 and R8 (in A and B). (**E** and **F**) *Eph* knockdown in R7 did not affect the separation of R7 and R8 growth cones and column formation (Ncad, blue). (**G**) Quantification of the size of R7 growth cones (in E and F). (**H**) Overlapping of the growth cones of R7 and R8 (in E and F). (**I** and **J**) R8-specific MARCM clones expressing *Eph* RNAi show the disorganization of R7 (Fas2, red) and R8 (*R8-Gal4 UAS-GFP*, green) growth cones. (**K**) Quantification of the size of R8 growth cones (in I and J). (**M** and **N**) R7-specific MARCM clones expressing *Eph* RNAi show no significant defect of R7 (*R7-Gal4 UAS-GFP*, green) and R8 (24B10, red) growth cones. (**L**) Quantification of the hole size of R8 growth cones (in I and J). (**O**) Quantification of the size of R7 growth cones (in M and N). (**P**) Quantification of the hole size of R8 growth cones (in M and N). Arrows indicate the clones. The dashed boxes are enlarged in the top-right panels (in A, B, E, F, I, J, M, and N). Scale bars, 5 μm.

Considering the expression pattern of Eph-Myc, Eph is most likely expressed in R8 and/or Mi1 (Fig. 2A). *Eph* knockdown in R8 induced morphological defects in R7 and R8 growth cones and in the column structure as visualized by Ncad (Fig. 4, A to D). This result is further supported by R8-specific MARCM clones expressing Eph RNAi showing the expansion of R7 growth cones, disorganization of R8 growth cones, and the overlapping of these growth cones (Fig. 4, I to L). Note that the specificity of Eph RNAi was verified by the previous rescue experiment (*34*). In contrast, *Eph* knockdown in R7 induced no significant defect (Fig. 4, E to H). The results of R7-specific Eph RNAi clones corroborated these observations (Fig. 4, M to P). In addition, *Eph* knockdown in Mi1 induced strong morphological defects in Mi1 whereas the growth cones of R8 were not significantly disrupted (fig. S5, C to G) suggesting that Eph is cell autonomously and nonautonomously required in R8 and is autonomously required in Mil for the organization of columnar neurons.

Overexpression of EphrinE320K (EphrinEK) in R8 resulted in disorganization of Mi1 growth cones, whereas overexpression of EphrinE320K in Mi1 had no significant impact on R8 growth cones (fig. S6). Together, these results indicate that Ephrin/Eph forward signaling between R7/R8 and R8/Mi1, as well as Eph/Ephrin reverse signaling between R8/R7, is crucial for columnar organization. Moreover, they suggest that bidirectional signaling is activated between R7 and R8.

### The interaction between Ephrin and Eph is required for Ephrin phosphorylation

**Fig. 5.**
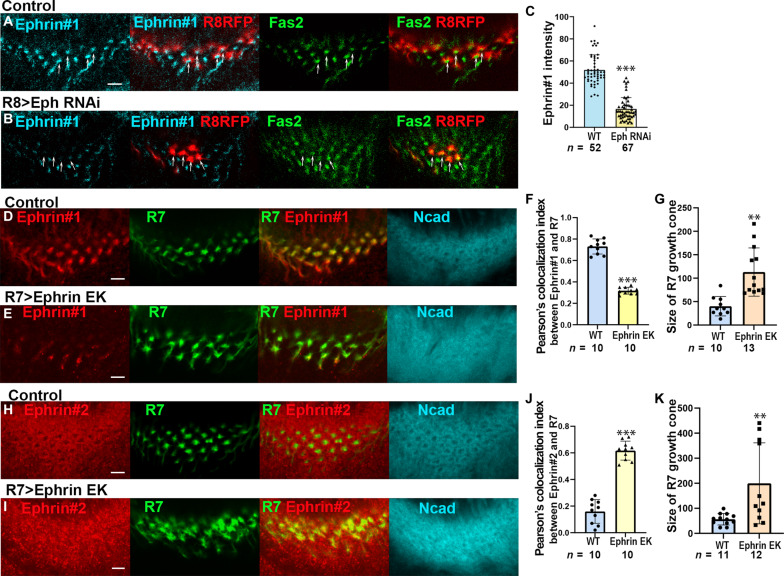
Interaction between Ephrin and Eph is required for Ephrin phosphorylation. (**A** and **B**) Ephrin#1 signals (blue) were down-regulated nearby R8 growth cones (*R8-LexA LexAop-RFP*) expressing *Eph* RNAi (white arrows). R7 growth cones were visualized by Fas2 staining (green). (**C**) Quantification of the signal intensities of Ephrin #1 (in A and B). (**D** to **K**) Expression of *Ephrin^EK^* in R7 caused down-regulation of Ephrin #1 signals (E), up-regulation of Ephrin #2 signals (I), and enlargement of R7 growth cones (E and I). (F and J) Quantification of the signal intensities of Ephrin #1 (in D and E) and Ephrin #2 (in H and I) in R7 growth cones. (G and K) Quantification of the size of R7 growth cones (in D, E, H, and I). Scale bars, 5 μm.

It is well established that Eph/Ephrin reverse signaling can be triggered by the binding of Eph ligand to Ephrin receptor on neighboring cells. To examine whether this mechanism may operate between R8 and R7 neurons, we generated Eph RNAi clones specifically in R8 (Fig. 5, A to C). Fas2 staining was used to label R7 growth cones. In this context, a reduction in the Ephrin #1 signal was observed, whereas the Fas2 signal remained detectable. Notably, the signal intensity of Ephrin #1, which may reflect the phosphorylated form of Ephrin, appeared to decrease upon Eph knockdown in adjacent R8 growth cones.

In addition, overexpression of *Ephrin^E320K^* (*Ephrin^EK^*) in R7 caused down-regulation of Ephrin #1 signals and up-regulation of Ephrin #2 signals in R7 growth cones, suggesting that the binding of Eph to Ephrin is required for Ephrin phosphorylation (Fig. 5, D to K). These findings are consistent with the idea that the interaction between Eph in R8 and Ephrin in R7 is crucial for Ephrin phosphorylation in R7.

### Tyrosine phosphorylation is required for Ephrin reverse signaling

**Fig. 6.**
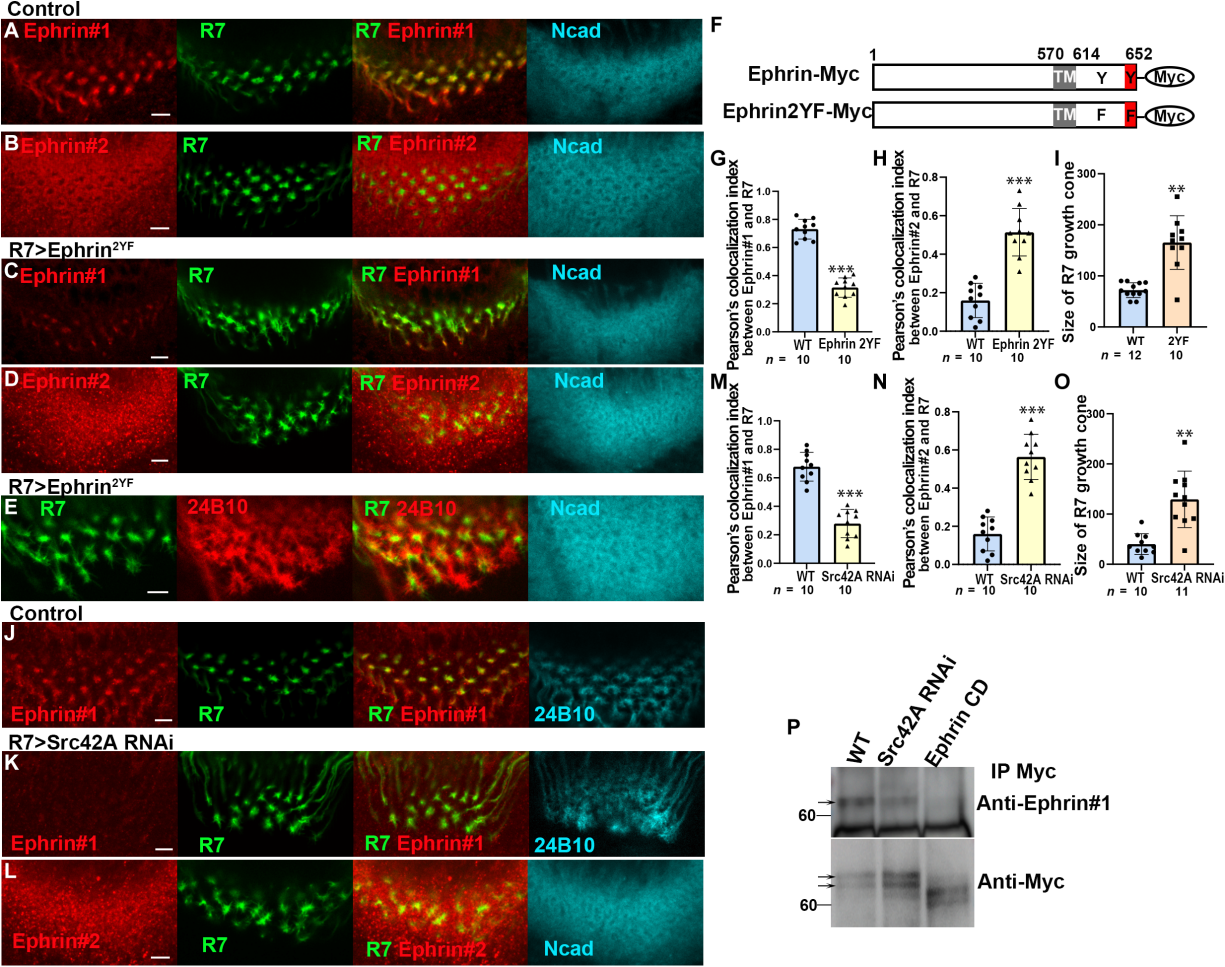
Tyrosine phosphorylation of Ephrin under the control of Src is required for Eph/Ephrin signaling. (**A** to **E**) Expression of *Ephrin^2YF^* in R7 decreased Ephrin #1 signals (C), increased Ephrin #2 signals (D), and enlarged the growth cones of R7 (B, D, and E). (**F**) Two tyrosine residues in the cytoplasmic domain of Ephrin were mutated to phenylalanine in *Ephrin^2YF^*. (**G** to **I**) Quantification of the signal intensities of Ephrin #1 (in A and C), Ephrin #2 (in B and D), and the size of R7 growth cones (in A to E). (**J** to **L**) *Src42A* knocked down in R7 decreased Ephrin #1 signals (K) and increased Ephrin #2 signals (L). 24B10, blue (J and K); Ncad, blue (L). (**M** to **O**) Quantification of the signal intensities of Ephrin#1 (in J and K), Ephrin #2 (in B and L), and the growth cones of R7 (in J and K). Results were statistically analyzed using Welch’s *t* test. **P < 0.001; ***P < 0.001; n.s., not significant. Scale bars, 5 μm. (**P**) Ephrin#1 signals were decreased upon *Src42A RNAi* and *EphrinCD* expression after immunoprecipitation.

To test whether Eph/Ephrin reverse signaling depends on tyrosine phosphorylation of Ephrin, we generated a mutant form of *Ephrin* in which the tyrosine residues in the cytoplasmic domain were mutated to phenylalanine (*Ephrin^2YF^*) (Fig. 6F). When Ephrin2YF was overexpressed in R7, the Ephrin #1 signal was decreased and the Ephrin #2 signal was increased (Fig. 6, A to D). In addition, R7 growth cones were expanded and disorganized, suggesting that tyrosine phosphorylation is required for Ephrin reverse signaling (Fig. 6, A to I). However, the tyrosine residues may not be the only amino acids that are phosphorylated in the cytoplasmic domain of Ephrin because the Ephrin #1 signal was more significantly decreased when EphrinCD was expressed in R7 than when Ephrin2YF was expressed (fig. S7). There is also a possibility that the increased Ephrin #2 signal observed in R7 growth cones may partially arise from growth cones of R8 and/or Mi1 that invade the center of the column.

### Src kinases are required for Ephrin phosphorylation

To identify tyrosine kinases that phosphorylate Ephrin, we performed R7-specific RNAi screening focusing on 12 genes encoding nonreceptor tyrosine kinases. We found that knockdown of *Src42A*, one of the two *Drosophila* Src homologs, decreased Ephrin #1 signals and increased Ephrin #2 signals in the growth cones of R7 axons (Fig. 6, J to O). The decrease in phosphorylated Ephrin was confirmed by Western blotting followed by immunoprecipitation from larval brain extracts (Fig. 6P). These results are consistent with the observation that Src positively regulates Ephrin-B phosphorylation and mediates Eph/Ephrin-B reverse signaling in vitro (*28*, *35*).

In *Drosophila*, Src42A and Src64B play redundant roles in multiple aspects of development (*36*–*38*). However, the Ephrin #1 signal was not significantly down-regulated upon *Src64B* knockdown in R7 (fig. S8, A and D). Because down-regulation of the Ephrin #1 signal was enhanced upon double knockdown of *Src42A* and *Src64B* (fig. S8, B to D), both are required for Ephrin phosphorylation, whereas Src42A plays more critical roles compared to Src64B. However, the uniform expression of Src42A in the medulla neuropil suggests the involvement of an unidentified effector in this process (fig. S8E).

### Fas2 is required for Ephrin phosphorylation and Eph/Ephrin reverse signaling

**Fig. 7.**
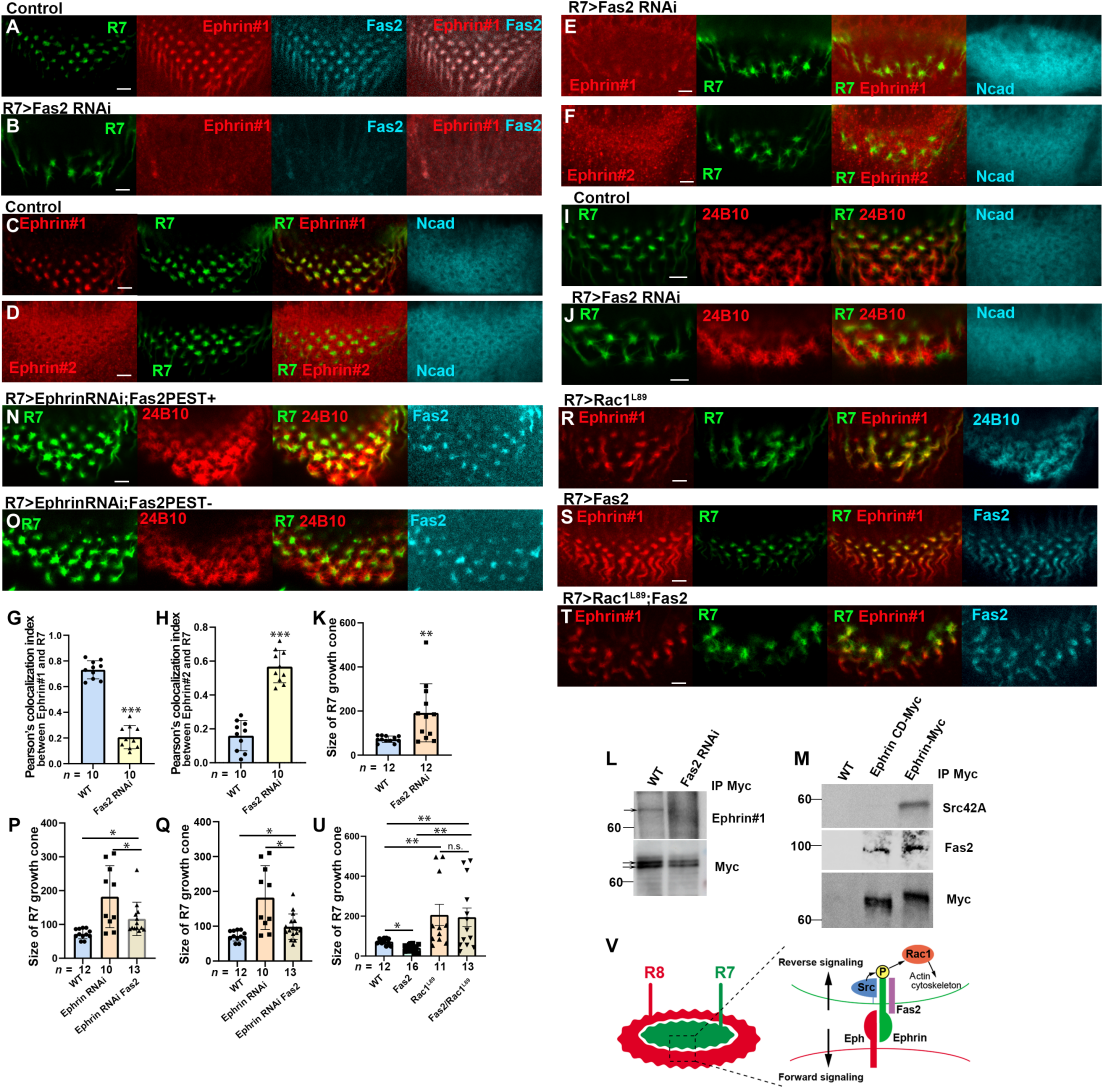
Fas2 is required for Ephrin phosphorylation in R7. (**A** and **B**) Down-regulation of Ephrin #1 signals (red) and Fas2 (blue) upon *Fas2 RNA*i in R7. (**C** to **F**) Down-regulation of Ephrin#1 (red in C and E) and up-regulation of Ephrin #2 signals (red in D and F) upon *Fas2 RNAi* in R7. Ncad, blue. (**G** to **K**) Quantification of the intensities of Ephrin #1 (in C and E) and Ephrin #2 (in D and F). (I and J) The growth cones of R7 (green) and R8 (24B10, red) were disorganized upon *Fas2 RNAi* in R7. (K) Quantification of the size of R7 growth cones (in I and J). (**L**) Ephrin#1 signals decreased upon *Fas2 RNAi* after immunoprecipitation (arrows). (**M**) Src42A and Fas2 were coimmunoprecipitated by Ephrin-Myc, whereas only Fas2 was coimmunoprecipitated by EphrinCD-Myc. (**N** and **O**) Simultaneous overexpression of Fas2 (PA+ in N or PA− in O isoforms) and Ephrin RNAi in R7 shows disorganized R7 (green) and R8 (red) growth cones. (**P** and **Q**) Quantification of the size of R7 growth cones (in P and Q). (**R**) The growth cones of R7 (green) and R8 (24B10, blue) were disorganized upon *Rac1^L89^* expression in R7. (**S**) Ectopic expression of *Fas2* (blue) in R7 reduced the size of R7 growth cones. (**T**) Ectopic expression of *Fas2* (blue) together with *Rac1^L89^* enlarged R7 growth cones. (**U**) Quantification of the size of R7 growth cones (in I and R to T). Scale bars, 5 μm. (**V**) Schematic drawing of Ephrin phosphorylation in R7 depending on the complex of Src, Fas2, and Ephrin triggered by Eph expressed in R8.

Fas2 is a member of the immunoglobulin superfamily and is homologous in structure and function to NCAM, a pivotal regulator of axon growth, fasciculation, and cell adhesion (*39*). It was reported that NCAM cooperates with the EphrinA/EphA system in restricting arborization of GABAergic interneurons in the mouse prefrontal cortex (*40*). However, it is not known whether NCAM and/or Fas2 is involved in Eph/Ephrin reverse signaling. We found that Fas2 is colocalized with Ephrin #1 signals in R7 (Fig. 7A). When *Fas2* was knocked down in R7, the Ephrin #1 signal was down-regulated and the Ephrin #2 signal was up-regulated, indicating that Fas2 is required for Ephrin phosphorylation (Fig. 7, A to H). The Fas2 dependence of Ephrin phosphorylation was confirmed by immunoprecipitation of Ephrin-Myc expressed under the control of *GMR-Gal4* followed by Western blotting (Fig. 7L). In addition, the R7 growth cones showed irregular morphology and larger size under the *Fas2* RNAi condition (Fig. 7, I and K). The column morphology was also disrupted as visualized by Ncad (Fig. 7, C to F, I, and J).

We observed Fas2 expression using the 1D4 antibody, which recognizes the intracellular domain of Fas2. The *fas2* gene is alternatively spliced to produce at least four protein isoforms. Two of these isoforms (PA+ and PA−), which differ in the presence or absence of the PEST domain, are transmembrane proteins that contain a cytoplasmic domain. Simultaneous overexpression of Fas2 (PA+ or PA− isoforms) and Ephrin RNAi in R7 partially rescued the phenotype observed under the Ephrin RNAi condition (Fig. 7, N to Q). Together, these data suggest that Fas2 is required for the Ephrin phosphorylation and Eph/Ephrin reverse signaling in R7.

Next, to verify whether Fas2 and Src42A interact with Ephrin, we performed a coimmunoprecipitation experiment in vivo. When Ephrin-Myc was immunoprecipitated with Myc antibody, coimmunoprecipitation of endogenous Fas2 and Src42A was detected by Western blotting (Fig. 7M). These results indicate that Src42A and Fas2 interact with Ephrin, which leads to Ephrin phosphorylation followed by Eph/Ephrin reverse signaling.

### Rac1 mediates Eph/Ephrin reverse signaling to control the organization of columnar neurons

Previous studies demonstrated that Ephrin-B reverse signaling plays a role in modulating axon retraction and pruning through a small guanosine triphosphatase (GTPase), Rac1/3 (*29*, *41*, *42*). To investigate the role of Rac1 in R7 growth cone organization, we expressed a dominant-negative form of Rac1 (Rac1^L89^) in R7, which resulted in the disorganization of the growth cones of R7 and R8, mimicking the effects observed when Ephrin or Fas2 function was impaired in R7 (Figs. 3B and 7, R and U). Notably, ectopic expression of Fas2 in R7 led to a reduction in the size of R7 growth cones compared to the control (Fig. 7, C, S, and U), suggesting that Fas2 promotes Eph/Ephrin reverse signaling, which, in turn, reduces the size of R7 growth cones.

To examine whether Rac1 acts as an essential effector downstream of Eph/Ephrin reverse signaling, we overexpressed Fas2 together with the dominant negative form of Rac1 in R7. Because the reduced R7 growth cones caused by Fas2 overexpression were suppressed by coexpression of Rac1 dominant negative, the results suggest that Rac1 acts downstream of Eph/Ephrin reverse signaling induced by Fas2 (Fig. 7, R to U). Thus, Rac1 acts as a downstream effector of Eph/Ephrin reverse signaling to regulate the organization of columnar neurons.

## DISCUSSION

In this study, we demonstrated that the bidirectional repulsive Ephrin/Eph signal shapes the columnar unit by organizing the morphology and segregation of columnar neurons in the fly brain. Furthermore, we found that the binding of Fas2 and Src kinase to Ephrin triggers tyrosine phosphorylation of Ephrin, which initiates Eph/Ephrin reverse signaling through Rac1. We presented a detailed analysis of the complex molecular interactions in the defined context of column formation.

During cerebral cortex development, different types of excitatory neurons originating from the proliferative ventricular zone radially migrate to form a cellular infrastructure of columns (*43*, *44*). Ephrin/Eph signaling was shown to be essential for columnar distribution and the proper assembly of cortical neurons (*29*, *30*). Similarly, in the fly brain, R7 and R8 axons project from the retina to the medulla where their growth cones comprise the medulla columns together with other axons and dendrites of columnar neurons such as Mi1. Our study proved that phosphorylated Ephrin and unphosphorylated Ephrin show distinct distribution patterns in columnar neurons. Ephrin in R7 was phosphorylated on the tyrosine residues under the control of Eph expressed in adjacent R8 to trigger Eph/Ephrin reverse signaling. Forward signaling of Eph in R8 and reverse signaling of Ephrin in R7 control the morphogenesis of the growth cones of core columnar neurons (Fig. 7V). Unphosphorylated Ephrin in R8 regulates forward signaling of Eph in Mi1 to control columnar neuron organization as well (figs. S4 and S5).

In vertebrates, transmembrane Ephrin-B acts as a receptor, in part mediated by its tyrosine phosphorylation. Ephrin-B has highly conserved tyrosine residues that are phosphorylated upon interaction with Eph (*45*). *Drosophila* Ephrin has a sequence homologous to Ephrin-B (*27*, *31*). Tyrosine and serine phosphorylation of Ephrin-B has been shown to function in two independent events regulating different aspects of Ephrin-B reverse signaling (*46*). In our study, tyrosine phosphorylation was shown to be required to regulate Ephrin reverse signaling by using a mutant form of Ephrin in which the two tyrosine residues in the cytoplasmic domain are mutated (Fig. 6).

We identified Src42A, one of the *Drosophila* SFKs, as a candidate that acts as a tyrosine kinase of Ephrin (Fig. 6, J to P). Ephrin phosphorylation and its reverse signaling was compromised by knocking down *Src42A* in R7. Consistent with this, SFKs were shown to positively regulate Ephrin-B phosphorylation and its phosphotyrosine-mediated reverse signaling in vertebrates (*28*). SFKs may be recruited to Ephrin-B in an Eph-dependent manner. We demonstrated the binding of Src42A to the cytoplasmic domain of Ephrin (Fig. 6P). Thus, the binding of Eph expressed in R8 to Ephrin expressed in R7 may lead to the recruitment of Src42A, which may induce the downstream event of Eph/Ephrin reverse signaling (Fig. 7N). Because the expression of Src42A is uniform in the columnar neurons (fig. S8E), we propose that there is an additional regulatory factor specifically expressed in R7 that controls the interaction between Src42A and Ephrin.

Fas2 is colocalized with phosphorylated Ephrin in R7. We demonstrated that Fas2 binds to the extracellular domain of Ephrin and is required for Ephrin phosphorylation (Fig. 7). Thus, Ephrin binds both Src42A and Fas2 in R7 resulting in Ephrin phosphorylation and activation of Ephrin reverse signaling triggered by Eph expressed in R8 (Fig. 7N). Fas2 may play a critical role in the formation of the complex of Ephrin/Src42A/Fas2 in the presence of Eph in adjacent cells. A previous study showed that NCAM, a homolog of Fas2, forms a complex with EphA3, which is necessary for EphrinA5/EphA3 signaling and plays a crucial role in restricting arborization of GABAergic interneurons in the mouse prefrontal cortex (*40*). However, its involvement in Eph/Ephrin reverse signaling was not known. On the basis of our data, Fas2 probably initiates the recruitment of Src42A to the Ephrin cytoplasmic domain under the control of Eph. In vertebrates, the PDZ-binding motifs in the cytoplasmic tail of Ephrin-B are involved in its tyrosine phosphorylation (*47*). However, the cytoplasmic tail of *Drosophila* Ephrin does not contain the PDZ binding sequence (*31*), suggesting that an alternative mechanism may induce Ephrin activation in *Drosophila*. Despite these differences, the tertiary complex of Ephrin/Src/NCAM (Fas2), or the quaternary complex including Eph, may play important roles in stabilizing Eph/Ephrin reverse signaling during column formation (Fig. 7N).

We next addressed a downstream effector of Eph/Ephrin reverse signaling that could regulate cytoskeletal organization at the growth cones of columnar neurons. The small GTPase, Rac1, was implicated in cell migration and neuronal morphogenesis through cytoskeletal organization (*48*–*50*) and was shown to be involved in Ephrin-B reverse signaling (*41*). We demonstrated that the effect of Fas2 overexpression in R7 to reduce the size of R7 growth cones was suppressed by coexpression of Rac1 dominant negative (Fig. 7), suggesting that Rac1 acts as a downstream effector of Eph/Ephrin reverse signaling to control the organization of columnar neurons.

Thus, we presented a comprehensive view of the complex molecular interactions in the defined context of column formation. It is highly likely that the molecular mechanisms demonstrated through our series of in vivo experiments are evolutionarily conserved from flies to mammals. Our findings open up an avenue of research that will help to broaden our understanding of the mechanisms of column formation and other related developmental processes.

## MATERIALS AND METHODS

### Fly strains

Standard *Drosophila* medium was used to maintain fly strains at 25°C. Both male and female flies were used for all experiments. The following fly strains were used as summarized in table S1 in accordance with the guidelines for transgenic animal experiments at Kanazawa University (KU6-1738): *UAS-Ephrin RNAi*, *UAS-Ephrin^E320K^*, *UAS-Eph-Myc*, *UAS-Eph RNAi*, *UAS-Ephrin40A RNAi*, *UAS-Eph40A RNAi*, *Ephrin ^I95^*, and *Eph^X652^* (from T. Chihara), *UAS-Src42A RNAi* (BDSC#44039), *UAS-Src64B RNAi* (BDSC#51772), *UAS-Fas2 RNAi* (BDSC#34084), *UAS-Rac1^L89^* (BDSC#6290), and *UAS-fas2PEST+ FLAG* and *UAS-fas2PEST-FLAG* (from O. Schuldiner) (*51*). *bshM-Gal4 UAS-myrGFP* (Mi1-GFP), *bshM-LexA LexAop-myrTomato* (Mi1-RFP), *sevEnS-Gal4 UAS-myrGFP* (R7-GFP), *sevEnS-LexA LexAop-myrTomato* (R7-RFP), *sensF2-Gal4 UAS-myrGFP* (R8-GFP), and *sensF2-LexA LexAop-myrTomato* (R8-RFP) were generated in our previous work (*3*). *sens-FLPase; GMR-FsF-Gal4 UAS-myrGFP* was used to perform R8-specific knockdown (*52*). R8/R7/Mi1-specific MARCM analyses were carried out crossing the following strains (Figs. 3, J to O, and 4, I to N, and figs. S4, J and K, and S5, E and F): *hsFLP; tubGal80 FRT40A; R7-/R8-/Mi1-Gal4 UAS-myrGFP* and *UAS-Eph RNAi FRT40A* or *UAS-Ephrin RNAi FRT40A*.

### Generation of *UAS-EphrinCD-Myc* and *UAS-Ephrin^2YF^-Myc* vectors and strains

The *Ephrin-Myc* plasmid was obtained as a gift from T. Chihara. *Ephrin-Myc* cDNA was amplified using forward primer *F1* and reverse primer *R1* and then inserted into the *NotI* and *KpnI* sites of the *pBSKS* vector. To create a cytoplasmic deletion of *Ephrin*, we used the *pBSK-Ephrin* vector as a template and carried out polymerase chain reaction (PCR) amplification using primer pair *CD_Fwd* and *CD_Rev.* The resulting PCR product was then self-ligated using the NEBuilder HiFi DNA Assembly Reaction (NEB) following the standard protocol. Similarly, we generated two-point mutations *Y650F* and *Y612F* of *Ephrin* using primer pairs *2YT_Fwd1/2YT_Rev1* and *2YT_Fwd2/2YT_Rev2*, respectively. The resulting PCR products were treated with *DpnI* (NEB) to remove the template plasmid DNA and then self-ligated using Ligation High Ver2 (Takara). All PCR products and enzyme-digested vectors were purified using the QIAquick PCR Purification Kit (Qiagen), and the resulting vectors were transformed into ECOS Competent *Escherichia coli DH5*α (Nippon Gene). After confirming the sequences, the cytoplasmic deletion of *Ephrin* and the two-point mutated *Ephrin* segments were excised using *NotI* and *KpnI* and reinserted into the *pUAST* vector. The resulting P-element vectors were then microinjected by GenetiVision (USA); *F1*: aattggagctccaccgcggccgcATTATGCAAGAACGATCAAAGCA, *R1*: gggaacaaaagctgggtaccCTAGACTAGTGGATCCCCCGG, *CD_Fwd*: attcgctctagacggTTCCATCGATTTAAAGCTATGGAGC, *CD_Rev*: tccgtctagagcgaatAAGATAGTGAATGCCAAGAATTGC, *2YT_Fwd1*: TTGACCGGTTCCATCGATTTAAAGC, *2YT_Rev1*: AATTCAATAGTGCCAGCATTC, *2YT_Fwd2*: TTAGTCCTGGAATGGTTGAA, and *2YT_Rev2*: AATCGCTGCACCGTGGTTTGC.

### Histochemistry

Immunohistochemistry was performed as described previously (*1*). Details are available upon request. Brains of wandering late third instar larvae, just before puparium formation, were dissected in phosphate-buffered saline (PBS), transferred to an ice-cold 0.8% formaldehyde/PBS solution, and fixed in 4% formaldehyde/PBS at room temperature for 30 to 60 min. The brains were washed in PBT (0.3% Triton X in PBS) and blocked in a 5 to 10% donkey normal serum/PBT solution at room temperature for 30 to 60 min. The primary antibody reaction was performed in a solution containing primary antibodies and 1% normal serum in PBT at 4°C overnight. The brains were washed in PBT. The secondary antibody reaction was performed in a solution containing secondary antibodies and 1% normal serum in PBT at 4°C overnight. After washing in PBT and PBS, the brains were mounted in VECTASHIELD (Vector Laboratories).

The following primary antibodies were used: rat anti-Ncad (1:20, Developmental Study Hybridoma Bank), mouse anti-Chaoptin (24B10, 1:20, Developmental Study Hybridoma Bank), mouse anti-Fas2 (1:20; Developmental Study Hybridoma Bank), rabbit anti-Myc (1:1000, Santa Cruz Biotechnology), rabbit anti-Src42A (1:200; from T. Kojima), and rabbit anti-Ephrin#1 and Ephrin#2 (1:1000; this work). Ephrin antibodies were generated in rabbits against the synthetic peptide, CNGMFDQNAGTIEYDR, which corresponds to the intracellular domain of Ephrin (WAKO, Japan; Fig. 2F).

The following secondary antibodies were used: anti-mouse Cy3 (1:200; Jackson ImmunoResearch, 715-165-151), anti-mouse Cy5 (1:200; Jackson ImmunoResearch, 715-175-151), anti-rat Alexa647 (1:100; Jackson ImmunoResearch, 712-605-150), and anti-rabbit Alexa546 (1:200; Invitrogen, A-11035). Confocal images were obtained by Zeiss LSM880 with Airyscan and processed using ZEN, Fiji, and Adobe Photoshop.

### Coimmunoprecipitation and immunoprecipitation

Dissected brain samples were collected in PBS and lysed with 1x lysis buffer containing 1× protease inhibitor cocktail (10x lysis buffer, Nacalai Tesque, Japan) and with or without phosphatase inhibitor (Nacalai Tesque) in the presence of 1 mM PMSF (phenylmethylsulfonyl fluoride) and 10 mM NaF. Anti-Myc-tag mouse antibody magnetic beads were added and immunoprecipitated according to the manufacturer’s protocol (MBL and Thermo Fisher Scientific). Western blotting was performed according to standard techniques. The following primary antibodies were used: rabbit anti-Ephrin#1 and Ephrin#2 (1:1000) and mouse anti-phosphotyrosine (1:1000; Sigma-Aldrich).

### Statistics and reproducibility

Distinct brain areas or samples were measured and analyzed as indicated below for quantification and statistical analysis. Results were statistically analyzed using two-sided Welch’s *t* test (*P < 0.05, **P < 0.001, and ***P < 0.001; n.s., not significant). Image intensities were not artificially processed, except as otherwise noted. At least six columns from more than 10 brain samples were examined. When statistics were not applicable, experiments were independently repeated at least three times with similar results.

### Image quantification

A custom MATLAB code was used to extract the *XY* coordinates of the growth cone terminals of R7 (https://doi.org/10.5281/zenodo.15803090). By manually selecting a hexagonal region of interest, a *Z*-projected image of a defined *Z*-interval was binarized using the Otsu method. Denoised by erosion and dilation, various image properties were extracted using MATLAB’s regionprops function. The coordinates of the centroids, the areas of the growth cone terminals, and the SD of the distance to the nearest centroid (irregularity of the R7 growth cone arrangement) were calculated.

Signal intensity was quantified within the indicated rectangle areas using Fiji. Coloc 2 function of Fiji was used to calculate the Pearson’s *R* value to quantify the colocalization between two different signals. The hole size of the R8 growth cone was quantified using the Oval function of Fiji. The graphs of quantified data were generated by Prism.
