# Supplementary material for: Src/Fas2-dependent Ephrin phosphorylation initiates Eph/Ephrin reverse signaling through Rac1 to shape columnar units in the fly brain
Source: Sci Adv. 2025 Aug 13;11(33):eadv7490. doi: 10.1126/sciadv.adv7490 (PMC12346307; doi:10.1126/sciadv.adv7490)
Supplement: Supplementary file 1 — Figs. S1 to S8 Table S1 [file sciadv.adv7490_sm.pdf]

Science Advances
AAAS

# Supplementary Materials for

## Src/Fas2-dependent Ephrin phosphorylation initiates Eph/Ephrin reverse signaling through Rac1 to shape columnar units in the fly brain

Miaoxing Wang *et al.*

Corresponding author: Makoto Sato, makotos@staff.kanazawa-u.ac.jp

*Sci. Adv.* **11**, eadv7490 (2025)
DOI: 10.1126/sciadv.adv7490

**This PDF file includes:**

Figs. S1 to S8
Table S1

Figure S1

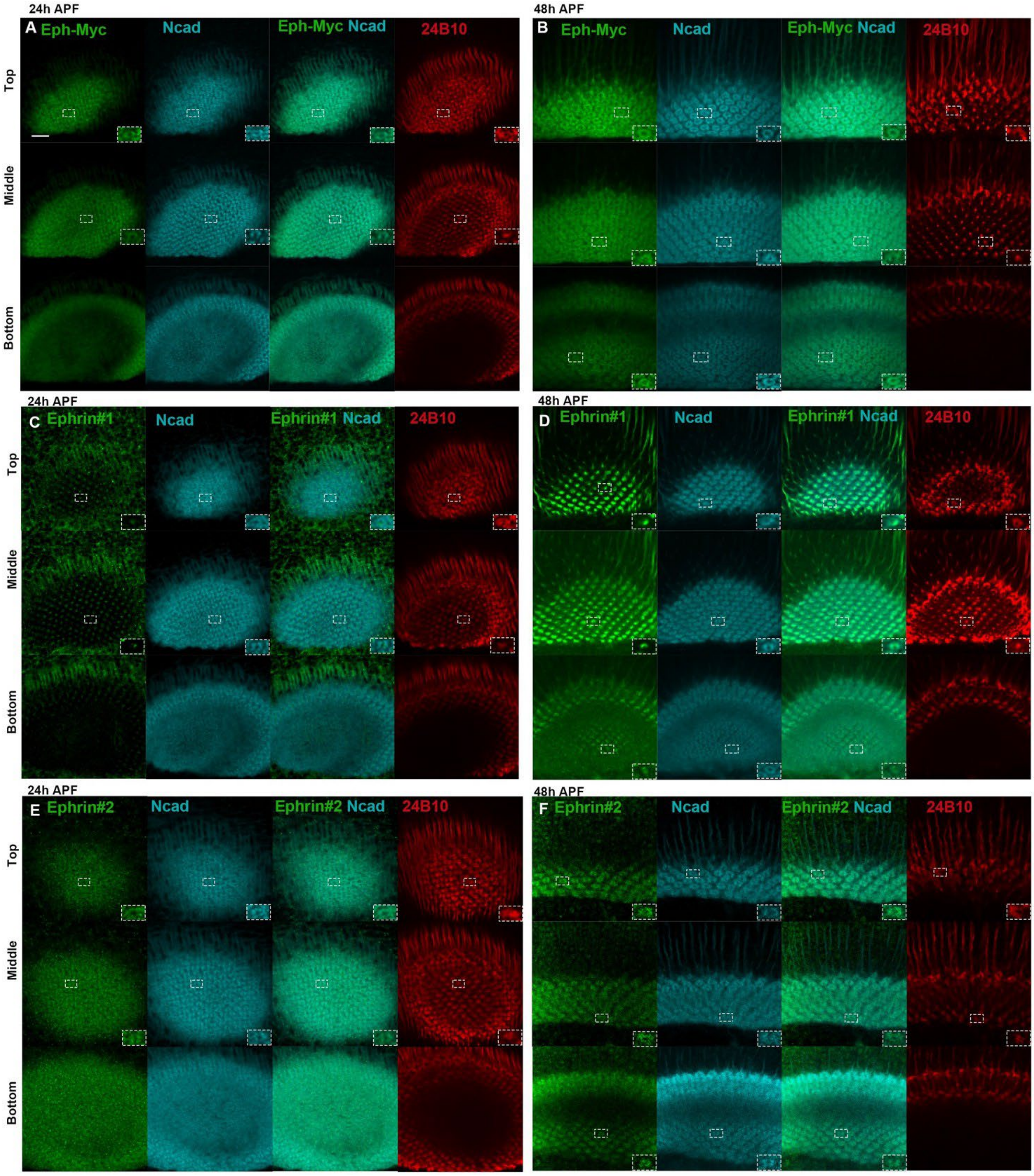


**Fig. S1. Eph and Ephrin distributions during pupal development.** (**A, B**) Eph-Myc shows a donut-like pattern (Myc, green) overlapping with Ncad (blue) at 24 h (**A**) and 48 h APF (**B**). (**C, D)** Ephrin #1 shows a dot-like pattern inside the donut-like domain of Ncad (blue) at 24 h (**C**) and 48 h APF (**D**). (**E, F**) Ephrin #2 shows a donut-like pattern overlapping with Ncad (blue) at 24 h and 48 h APF. 24B10 visualizes R7 and R8 growth cones (red). Scale bars, 5 μm.

## Figure S2

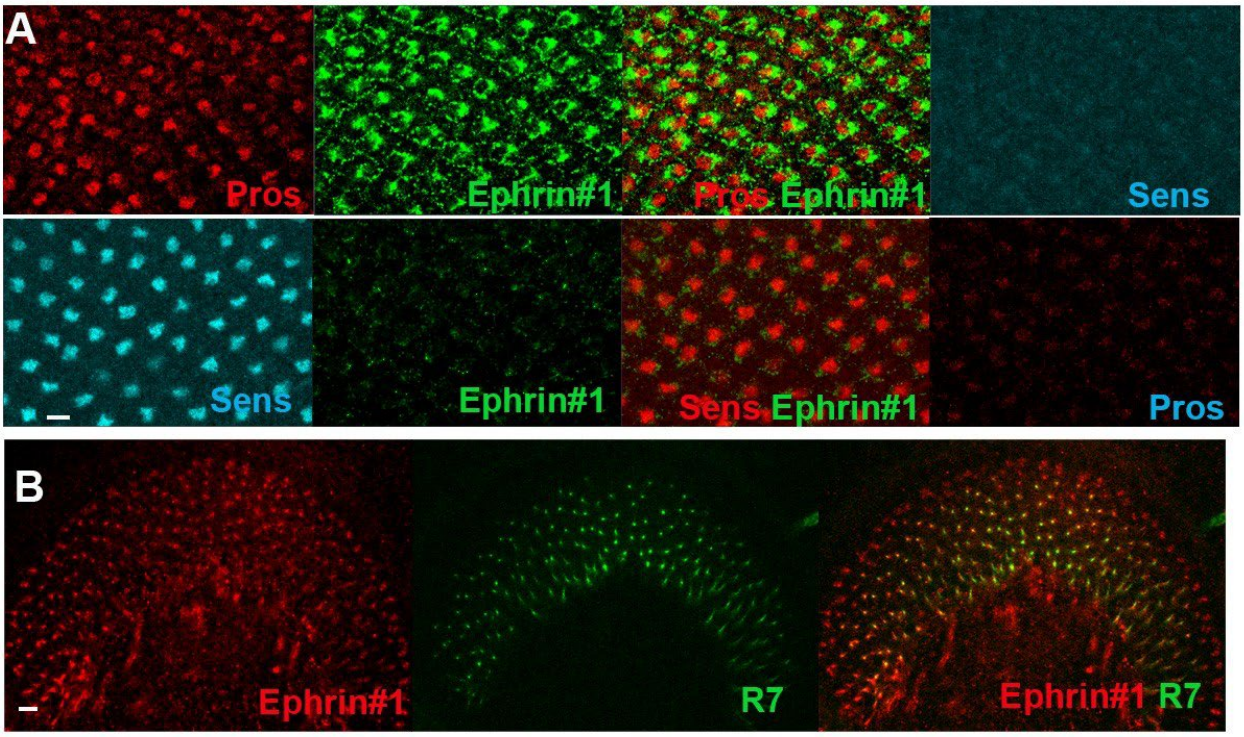


**Fig. S2. R7 is the source of Ephrin #1 signals.** (**A**) In the late third larval instar eye disc, strong Ephrin #1 signals are found in R7 cell bodies visualized by Pros (red), but not in R8 visualized by Sens (blue). Note that R7 and R8 cell bodies are located at different focal planes. (**B**) In the late third larval instar lamina, Ephrin #1 signals (red) overlap with R7 axons (green). Scale bars, 5 µm.

Figure S3

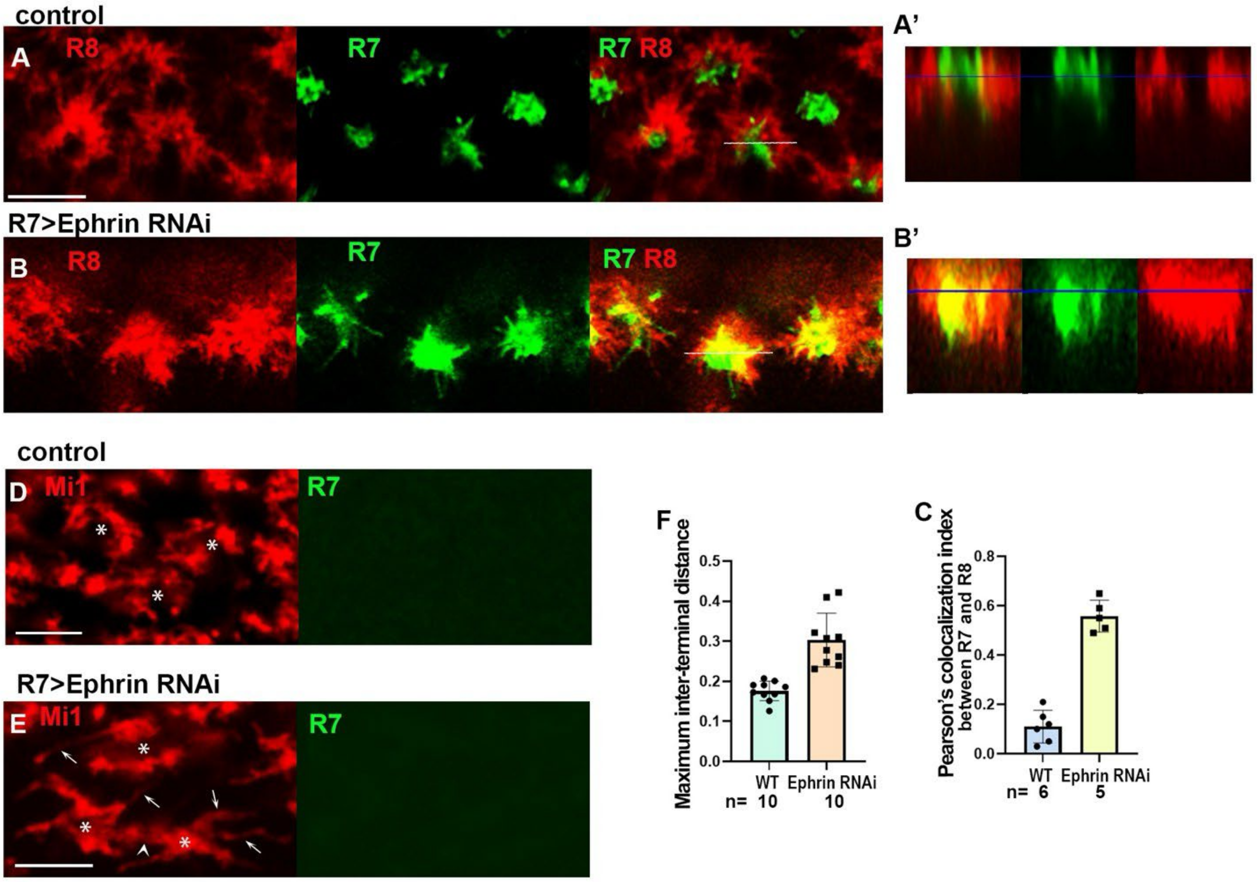


**Fig. S3. Ephrin expressed in R7 is required for column organization in larval and adult stages. (A, B)** *Ephrin* knock-down in R7 disturbs the separation of R7 (*R7-Gal4 UAS-GFP*, green) and R8 (*R8-LexA LexAop-RFP,* red) growth cones with super-resolution imaging (Zeiss Airyscan). **(A', B')** Lateral views along the lines indicated in (**A, B**) showing the overlap between R7 and R8 growth cones along the z-axis. (**C**) Quantification of the overlap between the growth cones of R7 and R8 (in **A, B**). (**D, E**) *Ephrin* knock-down in R7 (*R7-Gal4 UAS-GFP*, green) disturbs the morphology of Mil (*Mi1-LexA LexAop-RFP,* red) terminals in the adult. Asterisks indicate the centers of individual Mi1 terminals. Arrows indicate the elongated arborizations. Arrowhead indicates the terminal fusion. (**F**) Quantification of the maximum distance between arborizations in individual Mi1 terminals (in **D, E**). Scale bars, 5 µm.

Figure S4

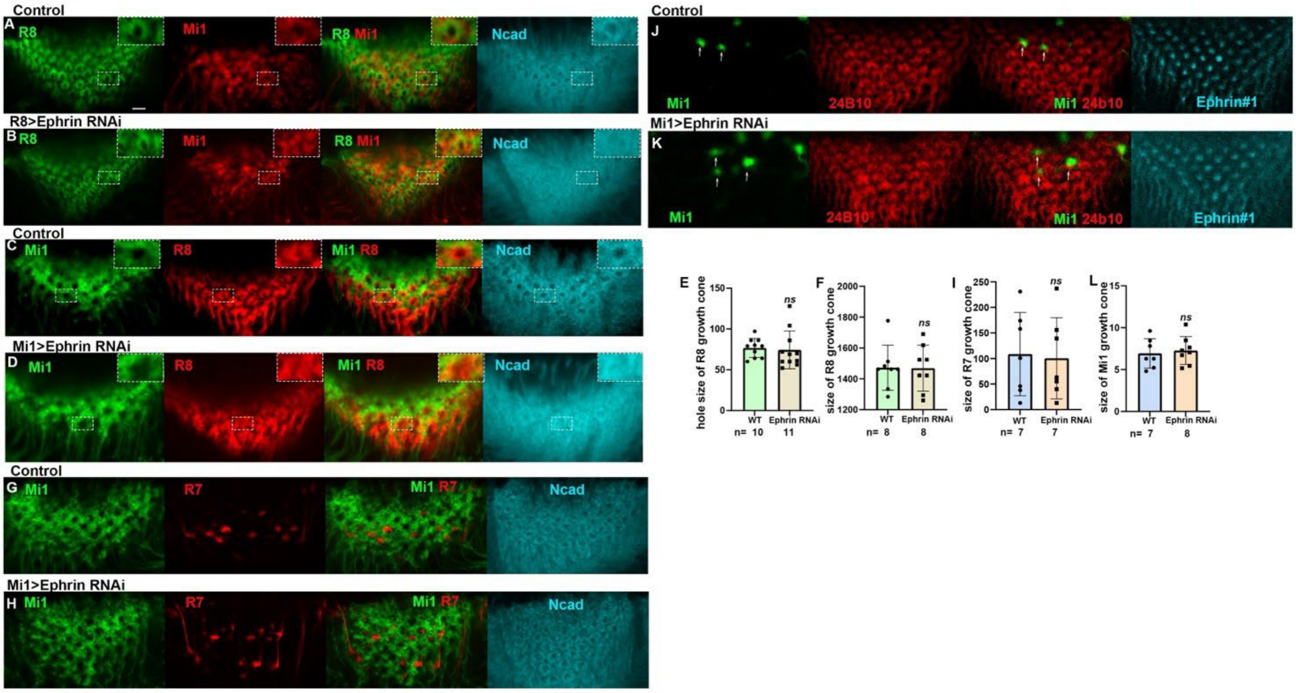


**Fig. S4. Ephrin expressed in R8 and Mi1 is non-autonomously required for column organization.** (**A, B**) R8 and Mi1 growth cones (*R8-Gal4 UAS-GFP*, green and *Mi1-LexA LexAop-RFP*, red) were disorganized upon *Ephrin* knock-down in R8. (**C, D**) R8 and Mi1 growth cones (*Mi1-Gal4 UAS-GFP*, green and *R8-LexA LexAop-RFP*, red) were not significantly disorganized upon *Ephrin* knock-down in Mi1. (**E**) Quantification of the hole size of R8 growth cones in **C** and **D**. (**F**) Quantification of the size of R8 growth cones in **C** and **D.** (**G, H**) R7 and Mi1 growth cones were not affected upon *Ephrin* knock-down in Mi1. (**I**) Quantification of the size of R7 growth cones in **G** and **H**. (**J, K**) Mi1-specific MARCM clones expressing *Ephrin* RNAi did not affect the organization of the Mi1 (*Mi1-Gal4 UAS-GFP*, green), R8 (24B10, red) and R7 (Ephrin#1, blue) growth cones. (**L**) Quantification of the size of Mi1 growth cones (in **J, K**). Arrows indicate the clones. n.s. not significant. Scale bars, 5 μm.

Figure S5

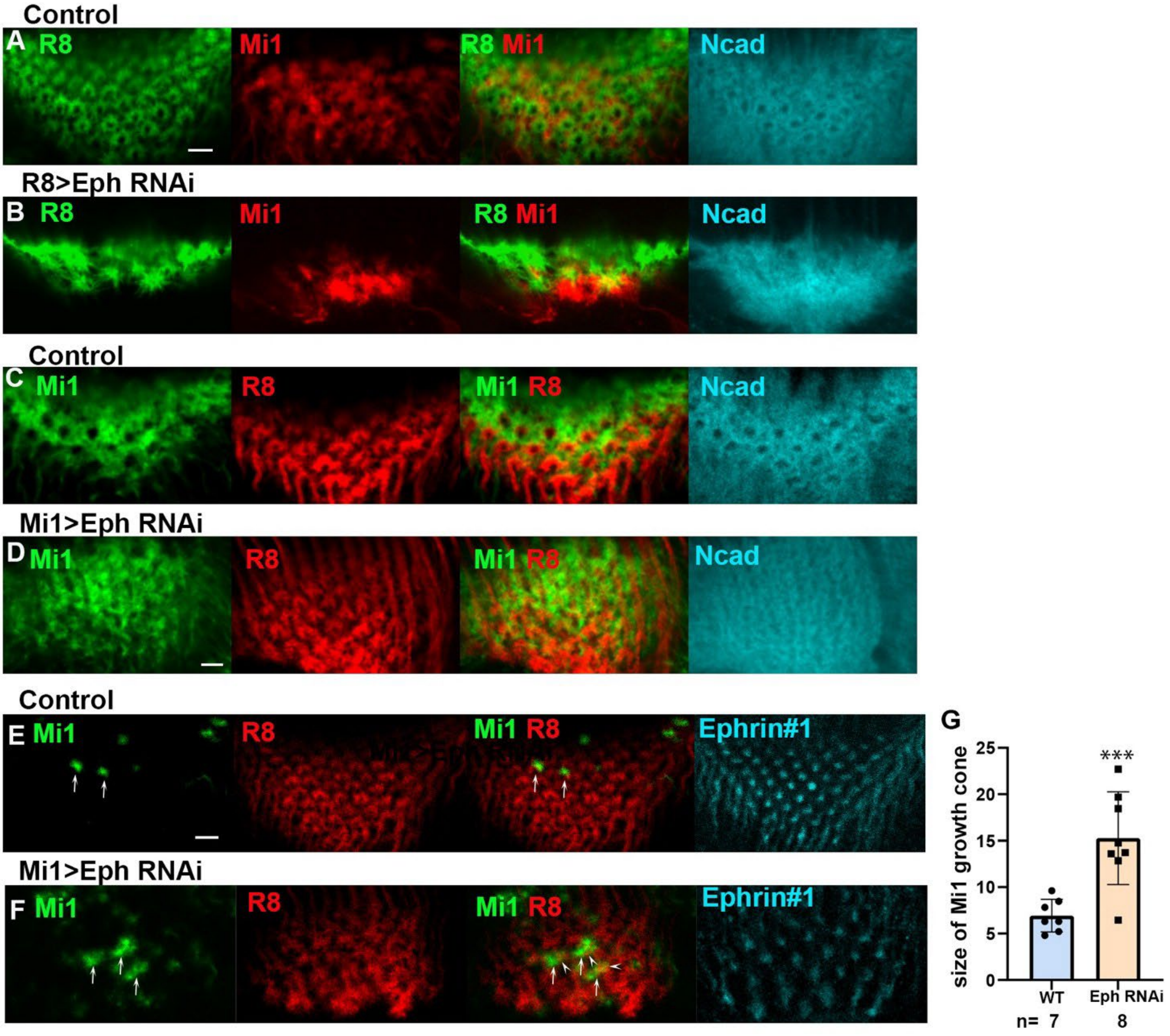


**Fig. S5. Eph expressed in R8 and Mi1 is required for column organization.** (**A, B**) R8 and Mi1 growth cones (*R8-Gal4 UAS-GFP*, green and *Mi1-LexA LexAop-RFP*, red) were disorganized upon *Eph* knock-down in R8. (**C, D**) R8 and Mi1 growth cones (*Mi1-Gal4 UAS-GFP*, green and *R8-LexA LexA-RFP*, red) were disorganized upon *Eph* knock-down in Mi1. (**E, F**) Mi1-specific MARCM clones expressing *Eph* RNAi show the spread growth cones of Mil that overlap with R8 (24B10, red), R7 shown by Ephrin#1 staining (blue). (**G**) Quantification of the size of Mi1 growth cones (in **J, K**). Arrows indicate the clones. Arrowheads indicate the spread of Mil growth cones overlapping with R8. Scale bars, 5 μm.

Figure S6

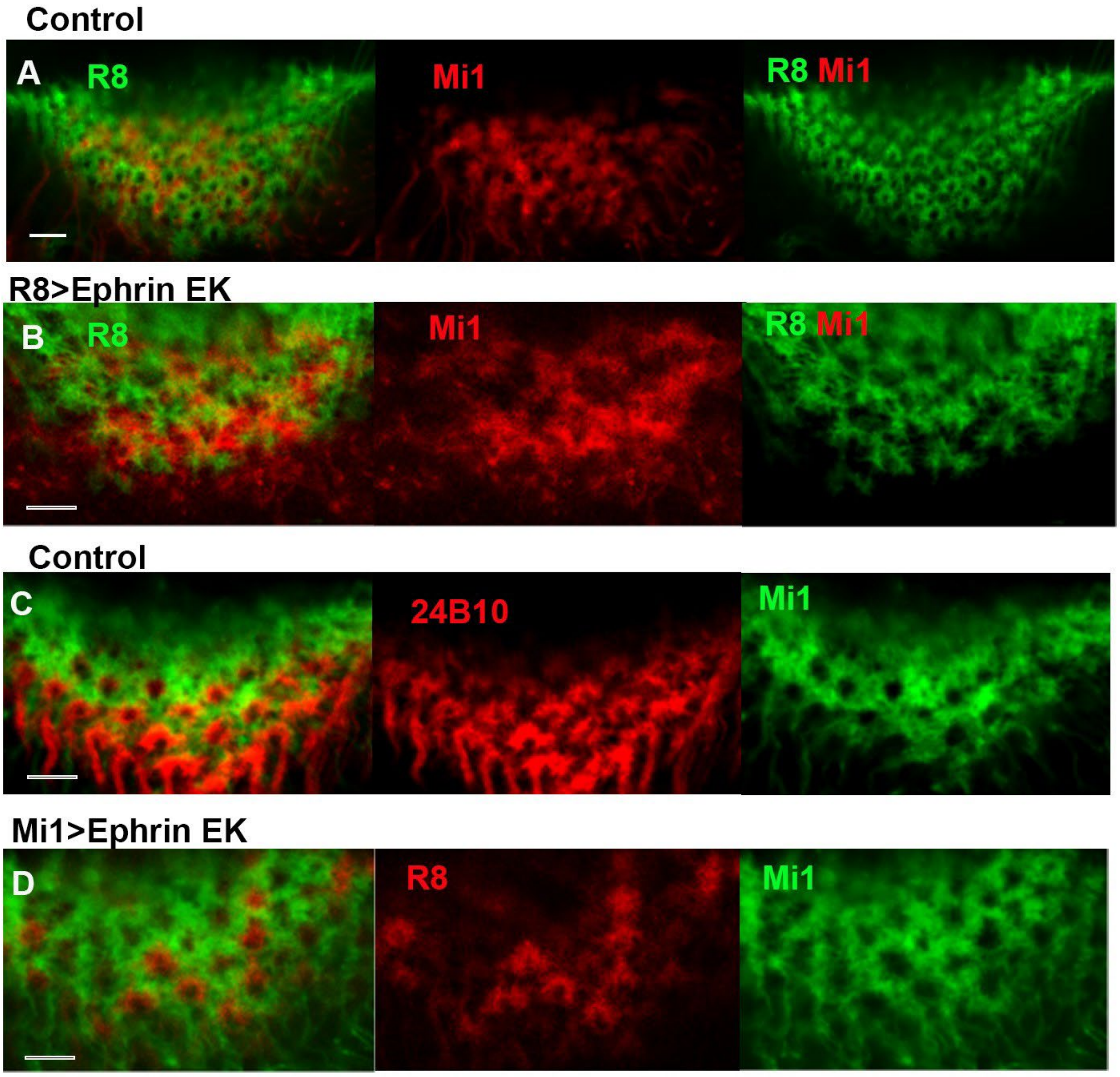


**Fig S6. Ephrin/Eph forward signaling between R8/Mi1 is crucial for columnar organization. (A, B)** Overexpression of EphrinE320K (EphrinEK) in R8 resulted in disorganization of Mi1 neurites. **(C, D)** whereas overexpression of EphrinE320K in Mi1 had no significant impact on R8 growth cones. Scale bars, 5 µm.

Figure S7

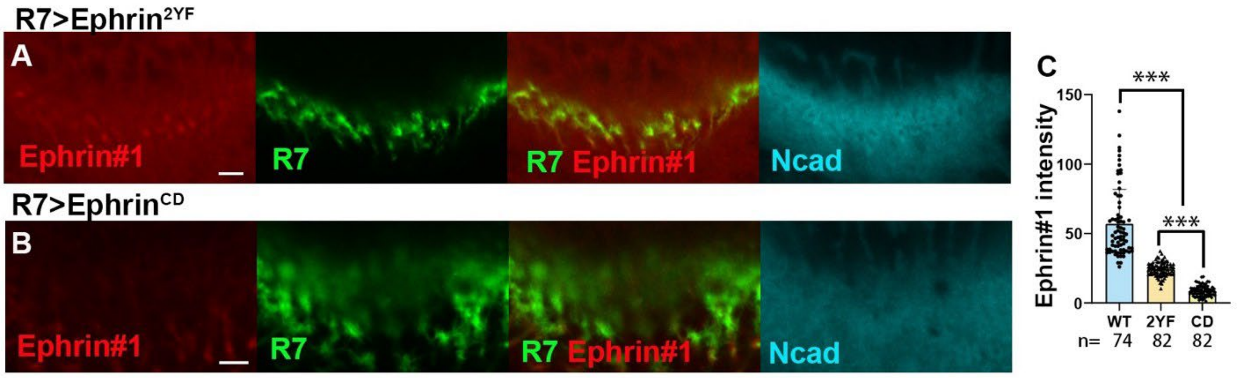


**Fig S7. Expression of mutant forms of *Ephrin* suppresses Ephrin phosphorylation.** (**A, B**) Ectopic expression of $Ephrin^{2YF}$ (**A**) and $Ephrin^{CD}$ (**B**) in R7 (*R7-Gal4 UAS-GFP*, green) reduces Ephrin #1 signals (red). See Figure 2I as a control. (**C**) Quantification of Ephrin #1 intensity. Scale bars, 5 µm.

Figure S8

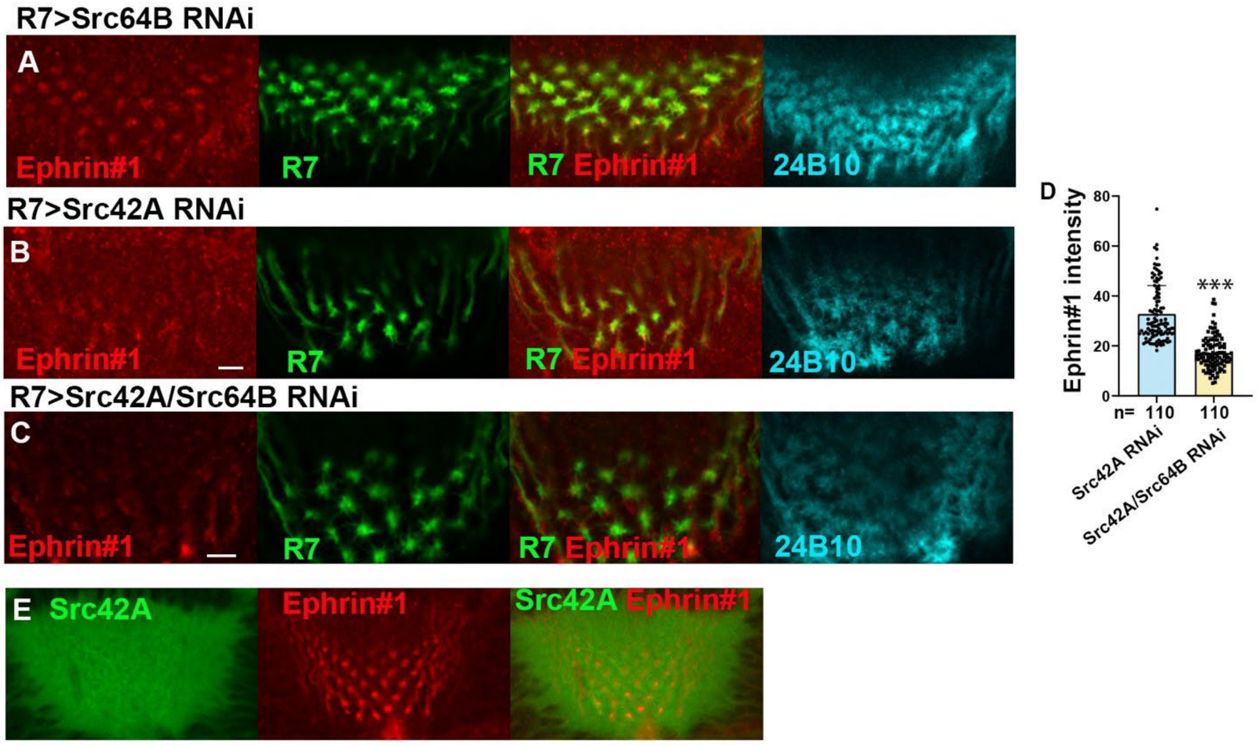


**Fig. S8. Src42A/Src64B double knockdown decreases Ephrin phosphorylation.** (**A**) *Src42A* knock-down in R7 (*R7-Gal4 UAS-GFP*, green) reduced Ephrin #1 signals. See Figure. 2I as a control. (**B**) *Src42A* and *Src64B* double knock-down in R7 (*R7-Gal4 UAS-GFP*, green) further reduced Ephrin #1 signals. (**C**) Quantification of Ephrin #1 intensity. Scale bars, 5 µm.

## Table S1

| Figure | Genotype | Source |
|---|---|---|
| Fig. 1 | *sevEnS-Gal4 UAS-myrGFP* (R7-GFP); | Trush et al., 2019 (3) |
| | *sensF2-Gal4 UAS-myrGFP* (R8-GFP); | Trush et al., 2019(3) |
| | *bshM-Gal4 UAS-myrGFP* (Mi1-GFP); | Trush et al., 2019 (3) |
| | *Ephrin* $^{I95}$ and *Eph* $^{X652}$ mutants. | Dr. Takahiro Chihara |
| Fig. 2 | *sevEnS-Gal4 UAS-myrGFP* (R7-GFP); | Trush et al., 2019 (3) |
| | *sensF2-Gal4 UAS-myrGFP* (R8-GFP); | Trush et al., 2019 (3) |
| | *bshM-Gal4 UAS-myrGFP* (Mi1-GFP); | Trush et al., 2019 (3) |
| | *UAS-Eph-Myc* | Dr. Takahiro Chihara |
| | *UAS-Ephrin RNAi* | Dr. Takahiro Chihara |
| | *UAS-EphrinCD-Myc* | Generated in this paper |
| Fig. 3 | *sevEnS-Gal4 UAS-myrGFP (*R7-GFP*);* | Trush et al., 2019 (3) |
| | *sevEnS-LexA LexAop-RFP (*R7-RFP*);* | Trush et al., 2019 (3) |
| | *sensF2-Gal4 UAS-myrGFP (*R8-GFP*);* | Trush et al., 2019 (3) |
| | *sensF2-LexA LexAop-RFP (*R8-RFP*);* | Trush et al., 2019 (3) |
| | *UAS-Ephrin RNAi FRT40A* | Dr. Takahiro Chihara |
| | *tubG80FRT40A* | BDSC#5192 |
| | *UAS-EphrinEK* | Dr. Takahiro Chihara |
| Fig. 4 | *sevEnS-Gal4 UAS-myrGFP* (R7-GFP); | Trush et al., 2019 (3) |

## Table S1

| Figure | Genotype | Source |
|---|---|---|
| | *sevEnS-LexA LexAop-RFP (*R7-RFP*);* | Trush et al., 2019 (3) |
| | *sensF2-Gal4 UAS-myrGFP* (R8-GFP); | Trush et al., 2019 (3) |
| | *UAS-Eph RNAi* FRT40A | Dr. Takahiro Chihara |
| | *tubG80FRT40A* | BDSC#5192 |
| Fig. 5 | *sens-FLPase; GMR-FsF-Gal4 UAS-myrRFP* | Trush et al., 2019 (3) |
| | *sevEnS-Gal4 UAS-myrGFP* (R7-GFP); | Trush et al., 2019 (3) |
| | *UAS-EphrinEK* | Dr. Takahiro Chihara |
| Fig. 6 | *sevEnS-Gal4 UAS-myrGFP* (R7-GFP) | Trush et al., 2019 (3) |
| | *UAS-Src42A RNAi* | BDSC#44039 |
| | *UAS-Ephrin2$^{YF}$-Myc* | Generated in this paper |
| | *UAS-EphrinCD-Myc* | Generated in this paper |
| Fig. 7 | *sevEnS-Gal4 UAS-myrGFP* (R7-GFP) | Trush et al., 2019 (3) |
| | *UAS-Fas2 RNAi* | BDSC#34084 |
| | *UAS-Rac1$^{L89}$* | BDSC#6290 |
| | *UAS-fas2PEST+ FLAG* | Dr. Oren Schuldiner |
| | *UAS-fas2PEST- FLAG* | Dr. Oren Schuldiner |
| Fig. S1 | *UAS-Ephrin-Myc* | Dr. Takahiro Chihara |
| Fig. S3 | *sevEnS-Gal4 UAS-myrGFP (*R7-GFP*);* | Trush et al., 2019 (3) |
| | *sensF2-Gal4 UAS-myrGFP* (R8-GFP); | Trush et al., 2019 (3) |

## Table S1

| Figure | Genotype | Source |
|---|---|---|
| | *bshM-LexA LexAop-RFP* (Mi1-RFP) | Trush et al., 2019 (3) |
| Fig. S4 | *sensF2-Gal4 UAS-myrGFP* (R8-GFP); | Trush et al., 2019 (3) |
| | *bshM-LexA LexAop-RFP* (Mi1-RFP); | Trush et al., 2019 (3) |
| | *bshM-Gal4 UAS-myrGFP* (Mi1-GFP); | Trush et al., 2019 (3) |
| | *sevEnS-LexA LexAop-RFP (*R7-RFP*);* | Trush et al., 2019 (3) |
| Fig. S5 | *sensF2-Gal4 UAS-myrGFP* (R8-GFP); | Trush et al., 2019 (3) |
| | *bshM-LexA LexAop-RFP* (Mi1-RFP); | Trush et al., 2019 (3) |
| | *bshM-Gal4 UAS-myrGFP* (Mi1-GFP); | Trush et al., 2019 (3) |
| Fig. S6 | *sensF2-Gal4 UAS-myrGFP* (R8-GFP); | Trush et al., 2019 (3) |
| | *bshM-LexA LexAop-RFP* (Mi1-RFP); | Trush et al., 2019 (3) |
| | *UAS-EphrinEK* | Dr. Takahiro Chihara |
| Fig. S7 | *UAS-Ephrin2$^{YF}$-Myc* | Generated in this paper |
| | *UAS-EphrinCD-Myc* | Generated in this paper |
| Fig. S8 | *sevEnS-Gal4 UAS-myrGFP (*R7-GFP*);* | Trush et al., 2019 (3) |
